# Combined quantitative and qualitative optical coherence tomography angiography biomarkers for predicting active neovascular age-related macular degeneration

**DOI:** 10.1038/s41598-021-97652-2

**Published:** 2021-09-10

**Authors:** Cherng-Ru Hsu, Tso-Ting Lai, Yi-Ting Hsieh, Tzyy-Chang Ho, Chung-May Yang, Chang-Hao Yang

**Affiliations:** 1 Department of Ophthalmology, National Taiwan University Hospital, No. 7, Chung-Shan South Rd, Taipei, Taiwan; 2 Department of Ophthalmology, Tri-Service General Hospital, National Defense Medical Center, Taipei, Taiwan; 3 Department of Ophthalmology, National Taiwan University College of Medicine, Taipei, Taiwan

**Keywords:** Retinal diseases, Predictive markers

## Abstract

To investigate choroidal neovascularization (CNV) characteristics after anti-vascular endothelial growth factor (anti-VEGF) therapy in patients with neovascular age-related macular degeneration by optical coherence tomography angiography (OCTA) and to assess the potential predictive role of combined qualitative and quantitative biomarkers for disease activity. Patients diagnosed with type 1 or type 2 CNV via multimodal imaging who had received anti-VEGF treatment were retrospectively reviewed. Qualitative and quantitative CNV responses on OCTA after serial injections were analyzed. The enrolled eyes were divided into two groups based on treatment intervals during follow-up, including an active group with less than 12 weeks intervals and a stable group with 12 weeks or longer intervals. Fifty-six eyes of 56 patients were included in the study. Twenty-seven eyes (48.2%) were classified as the “active group”, and 29 eyes (51.8%) were categorized as the “silent group”. Qualitative biomarkers of CNV showed significant differences between the two groups (branching capillaries: 48.1% vs 6.9%, *p* = 0.001; anastomoses and loops: 81.5% vs 13.8%, *p* < 0.001; peripheral arcade: 40.7% vs 10.3%, *p* = 0.013, and hypointense halo: 81.5% vs 41.4%, *p* = 0.002). A significantly higher vessel density was found in the active group (median 39.6% vs 30.5%, *p* = 0.003). “Anastomoses and loops” and “vessel density” predicted an active CNV status with a probability of 93.7% and achieved the best performance. The combination of two potential biomarkers of CNV on OCTA shows good discrimination for the prediction of recurrent exudation auxiliary to structural OCT that might associate with disease activity.

## Introduction

Age-related macular degeneration (AMD) is the leading cause of blindness in people over 50 years of age, accounting for 8.7% of all blindness worldwide^1^. Choroidal neovascularization (CNV) is a distinct feature of neovascular age-related macular degeneration (nAMD) and is characterized by the abnormal growth of blood vessels sprouting from the choroid through the Bruch’s membrane^2^. Conventional dye-based angiography has been considered the gold standard for diagnosis and classification of CNV. However, CNV lesions on fluorescein angiography (FA) or indocyanine green angiography (ICGA) are obscured by vessel leakage, which prevents the precise assessment of the microvascular morphological features. Moreover, these invasive procedures can result in systemic allergic reactions due to dye injection. Recently, optical coherence tomography angiography (OCTA) has been introduced to provide better visualization of the morphology of the retinal and choroidal vasculature in a noninvasive manner^3,4^. CNV shapes and morphological changes in response to treatment can be monitored by OCTA imaging. With the advent of anti-vascular endothelial growth factor (anti-VEGF) drugs, different therapeutic protocols, either a pro-re-nata (PRN) or a treat-and-extend regimen, have been advocated to improve the visual outcomes of nAMD^5,6^.

CNV shapes and morphological features, such as “sea fan” or “medusa,” tiny branching vessels, anastomoses loops, peripheral arcades, and perilesional hypointense halos have been indicated as active lesions^7–9^. Long filamentous linear vessels, large mature vessels, or “dead tree aspect” are mentioned in quiescent CNV^4,7^. Quantitative variables calculated using semiautomatic software to demonstrate the area, density, total vessel lengths, junction density, and lacunarity offered a reproducible, objective approach for CNV management^10^. Currently, simultaneously considering qualitative and quantitative OCTA biomarkers to improve diagnostic performance of disease activity has not been well established and discrepancies exist as to which biomarker is most representative of active lesion^11,12^.

This study aimed to identify minimal set of combined qualitative and quantitative OCTA characteristics that associates with recurrent exudative signs and its potential predictive role of disease activity following serial anti-VEGF treatments.

## Methods

We performed a retrospective observational cohort study of the medical records of patients observed at the National Taiwan University Hospital between September 2016 and October 2019. This study was approved by the Institutional Review Board of the National Taiwan University Hospital and conducted in accordance with the tenets of the Declaration of Helsinki.

### Study population

The baseline demographic characteristics of the patients including age, gender, best-corrected visual acuity (BCVA), central foveal thickness, previous ocular medical history, follow-up duration, and number of intravitreal injection (IVI) of anti-VEGF agents were recorded. The initial diagnosis of nAMD was established by FA and spectral domain OCT. All enrolled patients were treatment-naive or did not receive any treatment for at least 1 year before receiving the first anti-VEGF injection in this study and had been followed-up for at least 1 year. Exclusion criteria were presence of retinal vascular pathology other than nAMD, evidence of geographic atrophy or fibrotic scar, type 3 CNV, a refractive error greater than 6 diopters or axial length greater than 26.5 mm, and poor-quality images (scan image quality index < 5/10) due to severe motion, blinking artifacts, shadowing or projection artifacts on OCTA imaging. Patients were divided into two groups depending on their treatment intervals during follow-up after undergoing 2 or 3 monthly loading injections. Patients in the active group required anti-VEGF injection intervals less than 12 weeks. In the silent group, patients maintained dosing interval of twelve-week or longer. The OCTA examinations were performed at a minimum interval of 4 weeks after injections, and the OCTA images obtained after the last injections were used for the analysis. The treatment protocol and follow-up period received in our cohort were in accordance with the PRN regimen in the CATT study^13^ and was based on evidence of exudative signs (defined by the presence of intraretinal fluid, subretinal fluid, and/or subretinal pigment epithelial fluid) on structural OCT.

### OCTA image analysis acquisition

OCTA volumes of 3 × 3 mm were obtained at baseline and after treatments using the RTVue XR spectral domain OCT device (Optovue RTVue XR Avanti; Optovue, Inc., Fremont, CA, USA) via the split-spectrum amplitude decorrelation angiography algorithm. At a rate of 70,000 A-scans per second, the device produced two OCT volumes consisting of 304 × 304 A-scans each in approximately 2.6 s. The presence of neovascular complex was assessed on en face images generated by automatic segmentation obtained from the OCTA built-in software. Two horizontal segmentation lines set from the inner plexiform layer/ inner nuclear layer to the outer border of the Bruch’s membrane were used to visualize the CNV complex. The slab thickness was adjusted individually using the “Custom” function in the AngioVue to encompass the whole lesion best and ensure the least perilesional artifact based on CNV types and locations.

**Figure 1:**
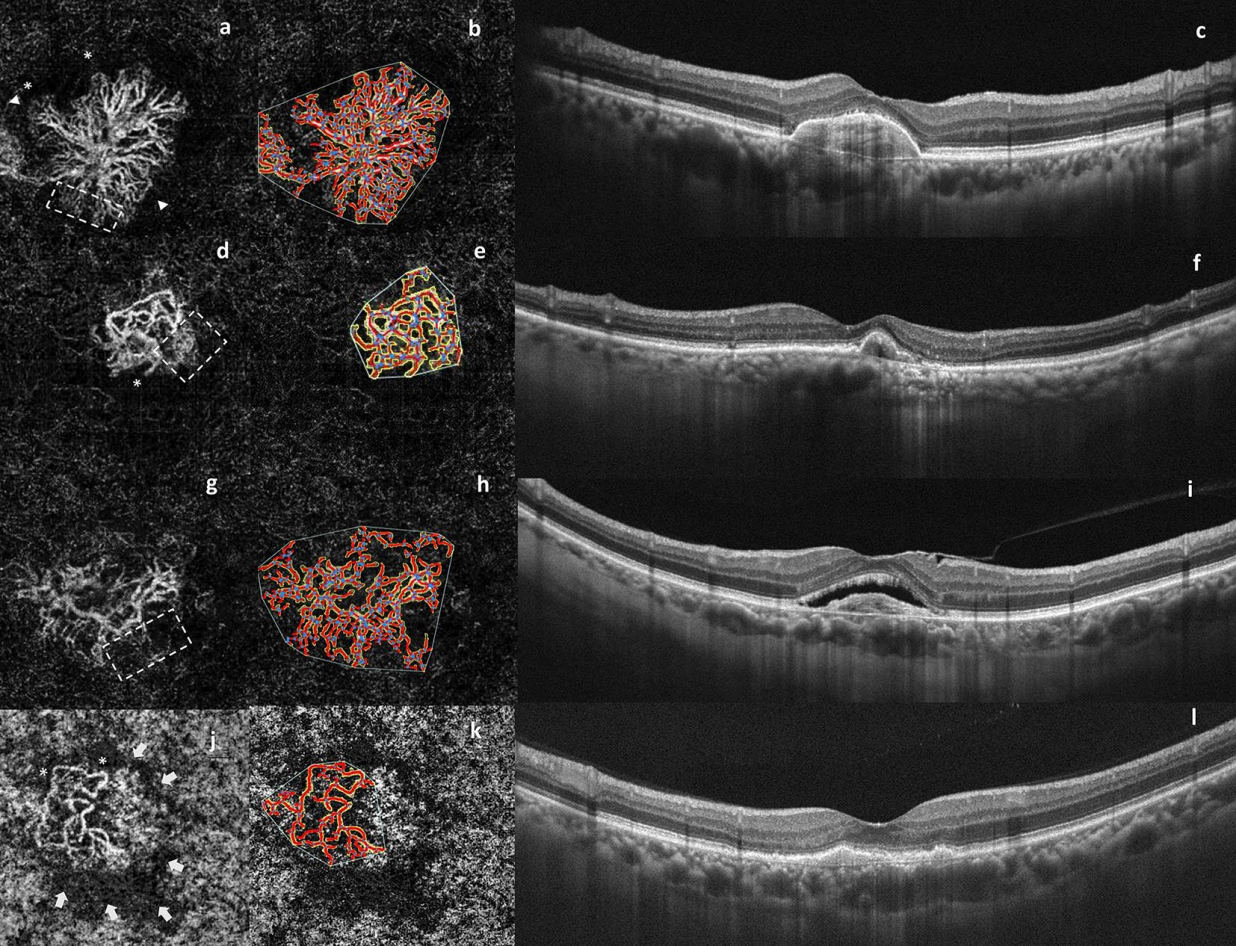
Representative examples of different choroidal neovascularization (CNV) shapes and their structural OCT scans after serial anti-VEGF treatments. (Left column) Medusa pattern, consisting of central feeder vessels with main vascular trunks and tiny capillaries that radiated from the center of the lesion in all directions on outer retina segmentation. Note the features of active lesions, such as branching capillaries (dotted square), anastomotic loops (asterisks), and peripheral arcade(arrow-heads) present after 15 intravitreal injections (**a**). Sea fan pattern with branching capillaries (dotted square) radiate from one side of the main vessel trunk and anastomotic loops (asterisks) of CNV on outer retina segmentation following four intravitreal injections (**d**). Indistinct pattern of CNV on outer retina segmentation with tiny branching capillaries (dotted square) are seen after six intravitreal injections. No anastomoses loops, peripheral arcade, or perilesional hypointense halo are noticed (**g**). Another indistinct pattern of CNV on the choriocapillaris slab showing anastomotic loops (asterisks) and a perilesional halo (arrows indicated area) following five intravitreal injections (**j**). (Middle column) The neovascular network is highlighted in yellow, automatically detected vessels are highlighted by red lines, and vessel junctions are highlighted by blue spots (**b**, **e**, **h**, **k**). (Right column) The corresponding OCT B-scans of the medusa shape CNV reveals subretinal hyperreflective material without subretinal fluid accumulation (**c**). The OCT B-scans for the sea fan shape CNV reveals pigment epithelium detachment (PED) at the macula (**f**). The corresponding OCT B-scans for the indistinct shape CNV revealed posterior hyaloid membrane and epiretinal membrane with subretinal fluid accumulation (**i**). The associated OCT scans reveals type 1 CNV below the pigment epithelium without exudative structural signs for another indistinct shape CNV (**l**).

The shape of the CNV complex was classified into three categories: (1) “medusa” or “sea fan” pattern, consisting of central feeder vessels with main vascular trunks and tiny capillaries that radiated from the center of the lesion. (2) “Long linear vessels” pattern without prominent capillary ramifications. (3) “Indistinct network” pattern, defined as visualization of only the main vascular trunks and thin branches without detectable feeder vessels. Other morphological variables that were used to describe CNV lesions included (1) tiny branching capillaries, (2) anastomotic loops, (3) peripheral arcades, and (4) perilesional hypointense halo. En face OCTA images were analyzed for quantitative features, such as CNV area, vessel density, vessel length, number of CNV junctions, and lacunarity, using a validated, semi-automated software (Angiotool 0.6a, https://ccrod.cancer.gov/confuence/display/ROB2) (Fig. 1)^14^. The qualitative and quantitative analysis were reviewed by two masked retina specialists to independently determined the presence of the morphologic features and consistency of software readings. Disagreements between interpretations were resolved by open adjudication of other senior retina specialists in the same hospital to reach a consensus.

### Statistics

SPSS statistical software version 26 (SPSS Inc, IBM Company, Chicago, IL) was used for statistical analyses. For descriptive statistics, the mean and standard deviation were presented for continuous variables. Percentages were calculated for categorical variables. Inter-group comparisons were performed using the chi-square test or Fisher’s exact test for categorical variables, while either the Student’s t test or the Mann–Whitney U test was used for continuous variables. Inter-observer agreement was computed using a Kappa coefficient for qualitative variables.

Univariate analysis was applied to qualitative and quantitative variables. Backward stepwise multivariate logistic analysis was then performed for the prediction of significant variables. The goodness of fit was assessed using the Hosmer–Lemeshow test, and the models’ performance was assessed by the Akaike information criterion (AIC). The area under the receiver operating characteristic (ROC) curve (AUC) was used to assess the discriminative ability of the logistic models. A *p* value < 0.05 was considered statistically significant.

### Ethics approval and consent to participate

This study was approved by the Ethics Committee and Institutional Review Board of National Taiwan University Hospital and adhered to the Declaration of Helsinki. Informed consent is waived due to the retrospective nature of the study and no identifiable details were present. (The Institutional Review Board of National Taiwan University Hospital, No. 202007057RIN).

### Consent for publication

Informed consent is waived due to the retrospective nature of the study and no identifiable details were present.

## Results

### Clinical characteristics and demographic data

Fifty-six eyes from 56 patients with nAMD met the inclusion criteria for analysis in the present study. The mean age of our cohort was 69.9 ± 8.2 years and 41 (73.2%) were male patients. Twenty-seven eyes (48.2%) were classified as the active group, whereas 29 eyes (51.8%) were categorized as the silent group. The mean logMAR BCVA at the time of diagnosis was 0.99 ± 0.48 (Snellen equivalent, 20/200) in the active group and 0.90 ± 0.47(Snellen equivalent, 20/160) in the silent group (*p* = 0.528). A significant difference was found in the mean BCVA at last follow-up between the two groups, with better BCVA in the silent group (Log MAR 0.89 ± 0.53, Snellen equivalent, 20/160 vs Log MAR 0.63 ± 0.39, Snellen equivalent, 20/86, *p* = 0.031). The mean central foveal thickness was thinner in the silent group (259.0 ± 104.12 µm) than in the active group (307.8 ± 131.74 µm). The average number of anti-VEGF injections received per patient was significantly higher in the active group (7.74 ± 4.07) than in the silent group (5.24 ± 3.18, *p* = 0.034). Mean interval for intravitreal injection in the active group and the silent group were 2.70 ± 1.17 months and 2.98 ± 1.81 months, respectively. No significant differences were noted in age, gender, BCVA at the time of diagnosis, type 1 or type 2 CNV, mean follow-up time, and mean IVI interval between the two groups. The demographic and clinical features among the groups are summarized in Table 1.

**Table 1:** Demographic and clinical characteristics of patients.

Variable	Active (n = 27)	Silent (n = 29)	P value^†^
Age, mean ± SD (years)	70.43 ± 8.23	69.55 ± 8.06	0.740
Gender (M)	17	24	0.064
Mean BCVA, mean ± SD (LogMAR) (Snellen equivalent)	0.99 ± 0.48 20/200	0.90 ± 0.47 20/160	0.528
Mean BCVA, mean ± SD (LogMAR), last follow-up (Snellen equivalent)	0.89 ± 0.53 20/160	0.63 ± 0.39 20/86	0.031*
Central foveal thickness(μm)	307.8 ± 131.74	259.0 ± 104.12	0.131
**CNV classification, n (%)**			
Type 1	17 (63.0)	20 (68.9)	0.474
Type 2	3 (11.1)	5 (17.2)	
Type 1 + 2	7 (25.9)	4 (13.8)	
Follow-up time, mean ± SD (months)	21.71 ± 9.20	22.40 ± 10.03	0.792
Anti-VEGF treatment, mean ± SD, n	7.74 ± 4.07	5.24 ± 3.18	0.034*
IVI interval, mean ± SD (months)	2.70 ± 1.17	2.98 ± 1.81	0.451

Continuous variables were presented as mean and standard deviation (SD).

*IVI* intra vitreal injection, *BCVA* best-corrected visual acuity, *LogMAR* logarithm of the minimum angle of resolution.

^†^Statistical analyses of age, BCVA, central foveal thickness, follow-up time, number of anti-VEGF treatment, and IVI interval were performed using Student’s t tests. Analyses of other variables were performed using chi-square test.

*p < 0.05 (bold numbers) was considered statistically significant.

### Optical coherence tomography angiography variable analysis

**Figure 2:**
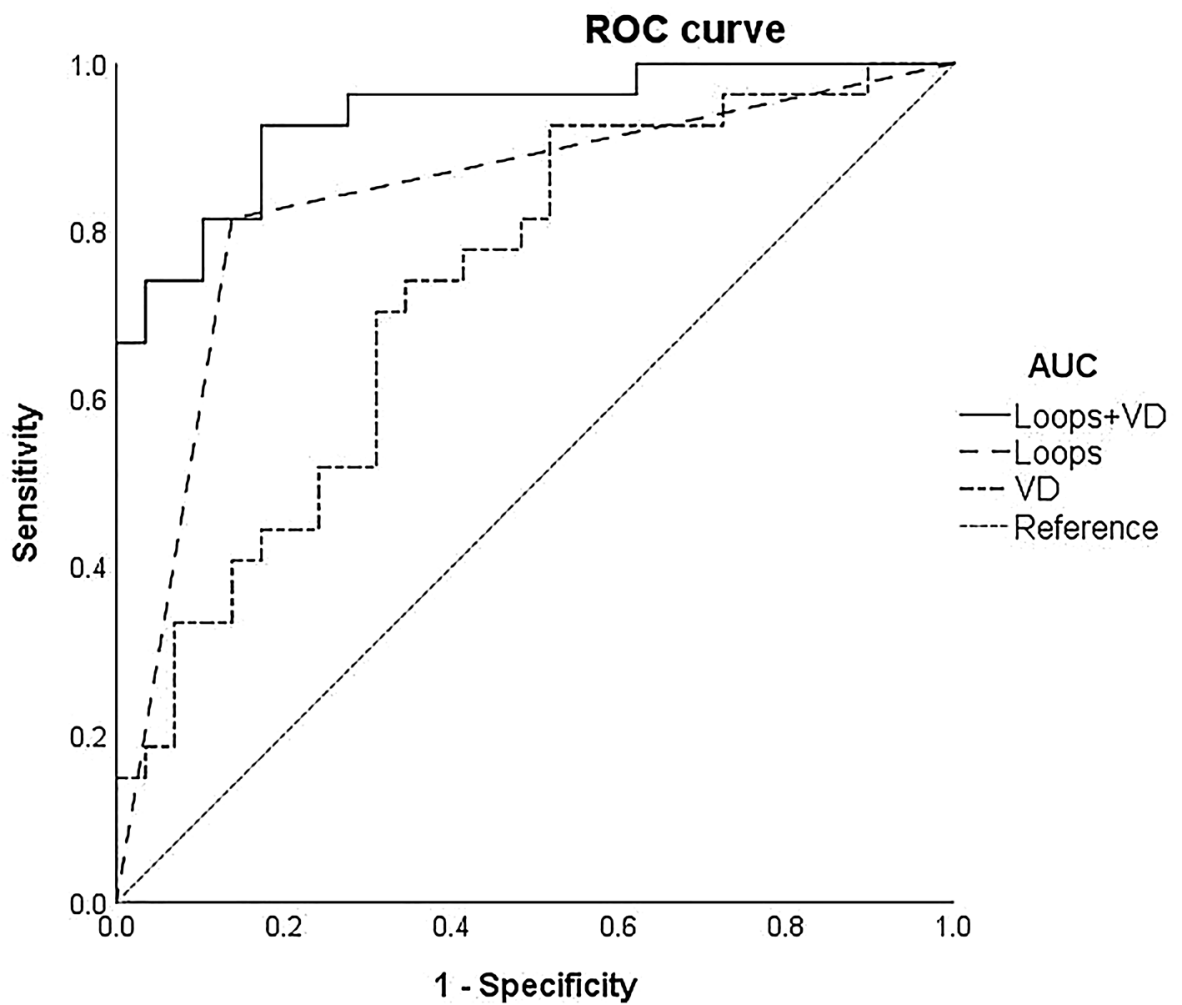
The receiver operating characteristic (ROC) curve between sensitivity and specificity revealed different area under the curve (AUC) of predicted models, with the highest probability of 0.937 for the correct prediction of choroidal neovascularization activity based on optical coherence tomography angiography biomarkers.

In en face OCTA analysis for the qualitative variables, an indistinct network was the most identified CNV shape among both groups (59.3% vs 59.6%) (Table 2). In the active group, the medusa or sea fan shape accounted for 8 eyes (29.6%), while the “long liner vessels” shape was observed in 11 eyes (36.9%) in the silent group. The four morphology variables, including branching capillaries, anastomoses and loops, peripheral arcade, and hypointense halo, were all found to be significantly more frequent in the active group than in the silent group (48.1% vs 6.9%, *p* = 0.001; 81.5% vs 13.8%, *p* < 0.001; 40.7% vs 10.3%, *p* = 0.013 and 81.5% vs 41.4%, *p* = 0.002, respectively). The kappa coefficients for inter-rater analysis revealed good agreement with 0.775, 0.732, 0.826, 0.779, and 0.888 for shapes, branching capillaries, anastomoses and loops, peripheral arcade, and hypointense halo, respectively. Of the quantitative parameters analyzed by the AngioTool software, a statistically significant difference (*p* = 0.003) was observed in vessel density, with higher density (median: 39.6%, IQR: 31.9–49.4%) in the active group than in the silent group (median: 30.5%, IQR: 21.6–40.0%) (Table 3). The median number of CNV areas of the active and the silent groups were 0.83 mm^2^ and 0.83 mm^2^ (*p* = 0.305), respectively. The median number of total vessel lengths of CNV in the active group and the silent group were 14.1 mm and 15.8 mm (*p* = 0.688), respectively. The median number of CNV junctions in the active group and the silent group was 53.0 and 52.0 (*p* = 0.617), respectively. The median number of lacunarity in the active group and the silent group was 0.29 and 0.32 (*p* = 0.204), respectively. No significance was found for the four variables. The sensitivity, specificity, and AUC for the potential qualitative and quantitative biomarkers for the active group after serial injections are shown in Table 4. In the multivariate logistic regression analysis, the presence of anastomoses and loops (*p* < 0.001) and the higher vessel density (*p* = 0.005) of CNV were biomarkers that were significantly associated with the active group (Table 5). The AUC of the ROC curve of the probability model from two variables in the backward analysis displayed good discrimination for the prediction of the active group (AUC = 0.937, *p* < 0.001) with the lowest AIC values. The AUC of the ROC curve for anastomoses and loops was 0.838 (*p* < 0.001), while that for vessel density was 0.743 (*p* = 0.003) (Table 6, Fig. 2).

**Table 2:** Optical coherence tomography angiography of qualitative parameters.

Variable	Active (n = 27, %)	Silent (n = 29, %)	*p* value^†^
**CNV shapes**			
Medusa or sea fan	8 (29.6)	1 (3.4)	0.007*
Long linear vessels	3 (11.1)	11 (36.9)	
Indistinct network	16 (59.3)	17 (59.6)	
Branching capillaries	13 (48.1)	2 (6.9)	0.001*
Anastomoses and loops	22 (81.5)	4 (13.8)	< 0.001*
Peripheral arcade	11 (40.7)	3 (10.3)	0.013*
Hypointense halo	22 (81.5)	12 (41.4)	0.002*

^†^Statistical analyses were calculated by chi-square test or Fisher’s exact test.

*p < 0.05 (bold numbers) was considered statistically significant.

**Table 3:** Optical coherence tomography angiography of quantitative parameters.

Variable	Active (n = 27)	Silent (n = 29)	*p* value^†^
CNV area, mm^2^	0.83 (0.61–1.43)	0.83 (0.36–1.18)	0.305
Vessel density (%)	39.6 (31.9–49.4)	30.5 (21.6–40.0)	0.003*
Total length of CNV, mm	14.1 (10.6–33.9)	15.8 (10.2–25.6)	0.688
Number of CNV junctions	53.0 (35.0–98.0)	52.0 (32.0–78.0)	0.617
Lacunarity	0.29 (0.26–0.36)	0.32 (0.26–0.44)	0.204

^†^Statistical analyses were calculated using the Mann–Whitney U test. Variables were reported as median and interquartile range (IQR)

*p < 0.05 (bold numbers) was considered statistically significant.

**Table 4:** Sensitivity, Specificity, and Diagnostic Accuracy of qualitative and quantitative parameters.

Variable	Sensitivity	Specificity	AUC^†^ (95% CI)
Branching capillaries	0.93	0.48	0.706 (0.57–0.85)
Anastomoses and loops	0.86	0.81	0.838 (0.73–0.95)
Peripheral arcade	0.90	0.41	0.652 (0.51–0.80)
Hypointense halo	0.59	0.81	0.701 (0.56–0.84)
CNV area, mm^2^	0.89	0.35	0.581 (0.43–0.73)
Vessel density (%)	0.93	0.48	0.733 (0.61–0.86)
Total length of CNV, mm	0.33	0.79	0.531 (0.38–0.69)
Number of CNV junctions	0.37	0.76	0.539 (0.39–0.69)
Lacunarity	0.96	0.28	0.599 (0.45–0.75)

^†^AUC: area under the receiver operating characteristic curve.

**Table 5:** Univariate and backward stepwise multivariate logistic regression analysis of qualitative and quantitative factors correlated with active group.

Variable	Univariate analysis		Multivariate analysis	
	OR (95% CI)	p value	OR (95% CI)	p value
**CNV shapes**		0.022*		
Long ve**s**sel versus medusa or sea fan	0.034 (0.0–0.4)	0.007*		
Indistinct versus medusa or sea fan	0.118 (0.0–1.0)	0.055	–	–
Branching	12.536 (2.5–63.5)	0.002*	–	–
Loops	27.500 (6.6–115.4)	< 0.001*	68.420 (8.6–381.5)	< 0.001*
Arcade	5.958 (1.4–24.7)	0.014*	–	–
Halo	6.233 (1.8–21.1)	0.002*	–	–
CNV area, mm^2^	1.581 (0.7–3.7)	0.286	–	–
Vessel density (%)	1.076 (1.0–1.3)	0.006*	1.137 (1.0–1.3)	0.005*
Total length of CNV, mm	1.010 (0.9–1.0)	0.554	–	–
Number of CNV junctions	1.005 (0.9–1.0)	0.464	–	–
Lacunarity	0.953 (0.9–1.0)	0.081	–	–

*p < 0.05 (bold numbers) was considered statistically significant.

**Table 6:** The AUC of the ROC Curve, sensitivity, and specificity of different predicted models.

Variable	AUC (95% CI)	Sensitivity	Specificity	AIC
Combined Loops and VD	0.937 (0.88–0.99)*	0.93	0.83	41.8
Loops	0.838 (0.73–0.95)*	0.86	0.81	51.4
VD	0.733 (0.61–0.86)^†^	0.93	0.48	70.1

*p < 0.001.

^†^*p* = 0.003.

loops = anastomoses and loops; VD = vessel density (%); AIC = Akaike information criterion.

## Discussion

The present study demonstrated different OCTA biomarkers correlated with the activity of CNV in patients with neovascular AMD undergoing PRN regimens and developed a probability model based on the combination of these pre-specified potential diagnostic qualitative and quantitative variables for recurrent exudative signs following serial anti-VEGF treatments. The highest probability of AUC and lowest AIC for sustained neovascular activity was reached in the presence of both one qualitative (anastomoses and loops) and one quantitative biomarker (vessel density) in contrast to only either a qualitative or quantitative parameter alone.

In our study, we found that 27 of 56 eyes (48.2%) remained in active status that required short injection intervals after mean 7.74 ± 4.07 anti-VEGF treatments, whereas 29 of 56 eyes (51.8%) progressed to a relatively inactive lesion after a mean of 5.24 ± 3.18 injections. A significant difference in BCVA improvement was noted between the two groups in the last follow-up, suggesting the attenuation of the CNV lesion without prominent subretinal scarring in the silent group. Although no statistical difference was observed in mean IVI intervals, the shorter intervals in the active group implied the nature of CNV lesions in this group was more active, and frequent injections were needed in response to fluid re-accumulation or persistent fluid. In contrast, the CNV lesions in the silent group behaved a more silent nature that tolerated relatively longer intervals for anti-VEGF treatment.

Recently, different biomarkers for evaluating clinically active and inactive CNV lesions on OCTA have been investigated to assess the anti-VEGF treatment response and to guide the treatment interval in patients with neovascular AMD. The combination of four qualitative biomarkers described by Coscas et al.^4^ showed a high probability for active lesion prediction in a previous study^15^. In our study, the sensitivity for branching capillaries, anastomoses and loops, peripheral arcade, and hypointense halo were 93.1%, 86.2%, 89.7%, and 58.6%, respectively. The specificity for branching capillaries, anastomoses and loops, peripheral arcade, and hypointense halo were 48.3%, 80.9%, 41.1%, and 81.4%, respectively. The probability of active CNV based on the presence of four biomarkers and CNV shape on OCTA was 89.3% in this study. In our cohort, CNV shapes and four qualitive biomarkers all demonstrated significant differences between the active and the silent groups. Most CNV lesions in our study were indistinct patterns. CNV shape assessment showed inconsistent results in its association with clinical activity, except that the presence of long filamentous linear vessels was associated with lesion inactivity^16^. Similarly, long linear vessels shape of CNV was significantly more in the silent group (36.9%) than in the active group (11.1%). The perilesional hypointense halo was the most frequent qualitative biomarker found among the two groups (81.5% and 41.4%, respectively) after serial anti-VEGF treatments. The halo seems to coincide with an area of low choriocapillaris flow that is closely parallel to CNV evolution^17^. Although regarded as an active qualitative biomarker, the presence of halo in the silent group might be associated with the remodeling of CNV, which allows better visualization of choroidal ischemia beneath the CNV itself. Anastomoses and loops were the second most frequent found biomarkers, which were compatible with the morphological changes proposed by Spaide that CNV evolves with vascular remodeling (arteriogenesis and angiogenesis) and is characterized by the prominent anastomotic connections of vessels and appearance of large-diameter vessels after serial injections^18,19^.

In contrast to the subjective assessments of qualitative biomarkers, we compared the quantitative biomarkers using the semiautomated Angiotool software to obtain a more objective analysis. OCTA provides reproducible imaging for the evaluation of the neovascular size in nAMD. Anti-VEGF therapy has been recognized to induce a quantitative regression with a variable decrease in size and vessel density of the neovascular membrane^9^ and high vessel density was considered as a feature of clinically active CNV^8^. The present data showed that both vessel density and CNV area were lower in the silent group, with the latter lacking statistical significance. These findings might reflect the better response to anti-VEGF in the silent group. One pilot study^20^ linked total vessel length to CNV activity after observing that it decreased significantly after the loading dose treatment. The mean total length of CNV was longer in the active group than in the silent group in our cohort but did not meet the level of significance. The number of CNV junctions could be interpreted as a measurement for vessel sprouting, with higher values being associated with active lesions^10^. Although no significant difference was noted, the number of CNV junctions was higher in the active group than in the silent group, which may indicate more active angiogenesis in the former group. Lacunarity is a parameter that describes the distribution of the sizes of gaps or lacunae surrounding the object within the image. In OCTA analysis, it represented a measure of CNV lesion homogeneity with higher values reflecting a more inhomogeneous vascular structure^10^. Since higher lacunarity was observed in quiescent lesions due to less homogenously filled space within the CNV lesion after anti-VEGF treatment, the lack of a significant difference between the two groups in our result may represent the subclinical activity of CNV in the silent group.

Using a logistic regression analysis for selecting final potential qualitative and quantitative biomarkers as a suitable model for discriminating highly active CNV lesions and assessing models’ overall performance by AIC, we propose two highly suggestive features, including one qualitative biomarker (anastomoses and loops) and one quantitative biomarker (vessel density) that showed high predictive power in combination, with an AUC of 0.937. The power of the predictive model for four qualitative biomarkers (AUC = 0.893) did not improve the probability as high as in a previous study^15^. In contrast to Coscas et al.^8^, modeling together the five quantitative biomarkers provided a relatively low AUC (0.840) in our study. Tiny vessel attenuation and pruned with a variable degree after anti-VEGF treatment might present a difficulty in reaching consensus between physicians when judging qualitative parameters on OCTA. The semiautomated analysis offered quantitative measurements that helped in objective comparison, which minimized the investigator bias for CNV activity. The application of present model auxiliary to structural OCT may help clinicians in identifying highly active nAMD with recurrent exudations following serial anti-VEGF treatments.

The limitations of our study include its relatively small sample size and retrospective cross-sectional design. We did not evaluate the qualitative and quantitative biomarkers longitudinally and thus lack information on its structural and microvascular changes after anti-VEGF treatment from the baseline. In addition, we did not enroll silent fibrotic lesions in our cohort, which may also affect the probability of predicting active CNV. Another limitation could be the inclusion of patients with different phases of angiogenesis between the two groups. Although the kappa coefficients revealed good agreement in our study, the branching complexity after vascular remodeling, shrinkage of peripheral vessels, and maturation of the remaining vessels after serial injections could still cause various degrees of morphological response that resulted in inconsistency in CNV interpretation. Furthermore, we excluded the undetectable hypointense CNV by OCTA which could have created a selection bias. The presence of RPE detachment or subretinal/intraretinal fluid could overlie the CNV and obscure vessel measurement. The treatment decision was based on exudative signs on structural OCT. Nevertheless, we present a model with combined OCTA parameters that adds value in predicting the recurrent exudation of nAMD.

In conclusion, OCTA is a noninvasive vascular imaging tool that is useful in detecting CNV morphology and blood flow characteristics as well as for assessing the CNV response after anti-VEGF treatment. The combination of the two potential qualitative and quantitative biomarkers identified on OCTA may signify the high possibility of sustained active neovascularization auxiliary to structural OCT. Larger prospective longitudinal studies are warranted to confirm the accuracy of the predicted model.

## Data Availability

All data generated or analysed during this study are included in this published article.
